# Misinformation and Junk Science in Obstetrics Medical Malpractice

**DOI:** 10.1097/og9.0000000000000073

**Published:** 2025-03-27

**Authors:** Michael G. Ross

**Affiliations:** Department of Obstetrics and Gynecology and Public Health, Geffen School of Medicine, the Fielding School of Public Health, UCLA, and the Institute for Women' and Children's Health, the Lundquist Institute, Los Angeles, California.

## Abstract

The practice of obstetrics continues to be influenced by concerns of medical–legal liability. Whereas assessments of clinical practice are commonly judged against society and community standards, issues of injury causation often are analyzed by a more complex scientific integration of anatomy, physiology, and pathophysiology. *Misinformation* refers to false or inaccurate information, especially that which is deliberately intended to deceive; *junk science* refers to scientific theories that are flawed, unreliable, or lacking in credibility. In the course of obstetric litigation, both clinical and scientific misinformation and junk science theories may be expounded in an attempt to bolster a case argument. These theories, though flawed, in their appeal to juries may have adverse consequences on clinicians and health care institutions and may unfortunately alter future clinical practice. Herein are presented several examples of misinformation and junk science theories that have been used to falsely attribute harm to the clinician. Together, medical societies and courts should aim to restrict the use of junk science.

## Abstract 2

Misinformation (ie, false or inaccurate information, especially that which is deliberately intended to deceive) and junk science (ie, theories that are flawed, unreliable, or lacking in credibility) have been advocated in obstetric medical malpractice suits, resulting in unsubstantiated allegations of fault and judgments against physicians and adversely affecting clinical practice.

Obstetrics is among the specialties at the highest risk for medical–legal liability, based on reports of both paid and closed medical liability claims.^1–3^ In part, this increased focus on obstetrics may be a consequence of the complexity of pregnancy issues, inherent risks of labor and delivery, the emotional nature of adverse maternal and neonatal outcomes, and the very high economic consequences of maternal or newborn injury. Although malpractice cases may bring an awareness and adherence to standardized medical practice by hospitals and physicians, greater tort liability was not demonstrated to improve quality of care.^4^ In contrast, there may be adverse effects of malpractice cases on physician practice as medical–legal concerns can affect clinical management, resulting in excessive and often unnecessary interventions^5^ (eg, cesarean deliveries) and excessive health care costs. Furthermore, malpractice cases may evoke emotional turmoil for both plaintiffs and defendants.

In all medical malpractice cases, the plaintiff typically must demonstrate both liability (ie, a breach of standard of care) and a causative attribution of care to the injuries. Liability and standard-of-care opinions from both defense and plaintiff experts can be based on society-established standards (eg, American College of Obstetricians and Gynecologists [ACOG] and Society for Maternal-Fetal Medicine), published literature, local or regional practice patterns, or the less reliable, “What I would have done.” On some occasions, with the motivation of plaintiffs’ attorneys to serve their clients' interests, a win-at-all-costs perspective may be used for both liability and causation arguments. In these occurrences, plaintiff experts may allege broad-based breaches of standard of care, involving a multitude of nurses and physicians, and theories of causation may be based on junk science.

*Misinformation* refers to false or inaccurate information, especially that which is deliberately intended to deceive, whereas *junk science* refers to scientific theories that are flawed, unreliable, or lacking in credibility.^6^ As opposed to standard-of-care controversies, theories of causation often are less familiar to the practicing clinician because they typically rely on scientific rather than clinical aspects of anatomy, physiology, and pathophysiology. Furthermore, complex mechanisms of causation may be even more challenging to the lay juror or judge. Junk science may be based on published literature or, alternatively, may be “creative” mechanistic interpretations. When based on published literature, the selected references may be poorly designed studies,^7^ biased interpretations,^8^ misinterpretation of data,^9^ studies that have not or cannot be replicated,^10^ and even reports published by individuals or groups with a potential bias.^11^ When based on creative interpretations, these references may invoke pseudoscience,^12,13^ a failure to adhere to the scientific method,^14^ or use of only select data that support a particular viewpoint (ie, reporting bias).^15^

Among the common flaws in junk science is the mistaken assumption of correlation for causation.^16^ Simply because two factors occur together, does not mean one was causative of the other. For example, one might question whether a neurologic condition caused an automobile accident or the automobile accident resulted in a neurologic condition. Alternatively, did two events simply occur at the same time? As a corollary to correlation versus causation, junk science may assume the post hoc fallacy that because one event precedes another, it must have caused the subsequent event. Even when a temporal sequence is established, it is not evidence alone of causation. Junk science theories may fail to consider confounding variables that may not be in a causative pathway, but can lead to erroneous conclusions regarding causation.^17^ Simply having risk factors for an adverse outcome does not demonstrate that the causative pathway is valid.^18^

In view of the common occurrence of these types of courtroom arguments, I present some examples of misinformation and junk science allegations within obstetrics.

## The Physician Failed to Deliver the Fetus Before it Depleted “Fetal Reserve”

The term “fetal reserve” is often used as a nonquantitative, undefined concept to suggest the capacity of the fetus to tolerate hypoxic stress. It is generally not used clinically to describe the effect of labor on a fetus. The term appears to originate from the study by Barron in 1953,^19^ in which he described a comparative study of the “alkali reserve” of normal, pregnant, and fetal sheep. In effect, Barron was describing the fetal base deficit or base excess as the ability to buffer acidosis. Numerous “reserve” articles followed this one, in which authors reported on the fetal alkali reserve. However, the ambiguity of “fetal reserve” is evident by subsequent studies referring to “fetal iron reserve,”^20^ “newborn carbohydrate reserve,”^21^ “serum albumin reserve”^22^ “fetal receptor reserve for acetylcholine,”^23^ “glycogen reserve,”^24^ and “fetal cardiac reserve,”^25^ among others. A 1975 publication apparently adopted the terminology of “fetal reserve”^26^ as the fetal response to spontaneous or induced uterine contractions. In this article, “fetal reserve” was, again, nonquantitative and nondefined with assessment of fetuses as simply positive or negative. As such, there is no accepted definition of fetal reserve. If anything, the origin of “fetal reserve” refers to base deficit,^19^ a value that can be accurately quantified by analysis of blood gases. In discussion forums, fetal reserve may be referred to in a general sense as the concept of the fetus' ability to tolerate periods of oxygen deprivation.

As background, among the criteria of neonatal signs consistent with an acute peripartum on intrapartum event, ACOG has defined *fetal umbilical artery acidemia* as a pH less than 7.0 or base deficit greater or equal to 12 mmol/L, or both. Whereas arterial pH values reflect a mix of both respiratory and metabolic acidosis, base deficit represents the component of metabolic acidosis. In contrast to these measurable and reportable pH and base deficit values, the term fetal reserve index has been proposed as a tool to better predict the potential for hypoxic injury during labor.^27–34^ Although a full discussion of the strengths and limitations of the fetal reserve index is beyond the scope of this report, there are several key issues that must be recognized. In the original article^31^ that proposed the fetal reserve index, the authors indicated a detailed risk-scoring system to calculate fetal reserve index but failed to provide the risk score weights. Remarkably, without supporting data, one author claimed the ability to identify the precise point (“point B”) on the fetal monitor tracing at which fetal neurologic injury occurred, using this point as evidence of the value of fetal reserve index. It should be noted that all fetal heart tracings in this publication were derived from cerebral palsy malpractice litigation cases. Of note, only 3–5% of neonates with umbilical artery base deficit 12–20 mMol/L develop cerebral palsy.^35^ Thus, the use of only cases that resulted in neurologic impairment represents a very select subgroup of children demonstrating newborn acidosis. To date, there has been no report of blind assessment of randomly obtained fetal tracings to determine whether one can confirm that an injury occurred, let alone the precise timing of the injury. More importantly, among these cases of alleged intrapartum hypoxic injury, only 35% demonstrated pH of less than 7.0, only 44% demonstrated Category III tracings, and only 30% met ACOG criteria for intrapartum hypoxic injury.^33^

In a subsequent report attempting to validate the fetal reserve index, the authors used selected cases from the 1970s in which repeated scalp blood sampling was performed.^32^ Notably, the median base deficit at 4 min of life was 11 mMol/L, a value far more acidotic than normal.^36^ In a further report^33^ alleging that the fetal reserve index has improved predictability compared with ACOG criteria, 12.5% of randomly selected, high risk cases that entered labor with reassuring tracings had umbilical cord blood based excess of less than −12 mMol/L.^33^ This level of acidosis is surprising, because it was reported that these cases were randomly selected and management supervised by “experienced faculty.” In each of these reports, cases and controls are minimally described as to management, interventions, institutions, and outcomes.

Many of the reported cases that failed to meet acidosis or ACOG criteria for intrapartum hypoxic brain injury are likely prelabor injuries, because the proportion of cerebral palsy associated with intrapartum hypoxia-asphyxia is reported to be less than 15%.^37^ To claim that the ability to pinpoint the time of neurologic injury with a proposed “patented” fetal assessment tool, which has never been tested in blinded manner, can establish a standard of care raises grave concerns for obstetric liability. As discussed above, the fetal reserve index represents publications by individuals or groups with a demonstrated bias.

## Excessive Physician Traction is the de Facto Cause of Permanent Brachial Plexus Injuries

In the late 1900s, the primary risk factor attributed to brachial plexus injury, with minimal evidence, was excessive physician lateral or axial traction.^38^ During the past 20 years, however, data have demonstrated that maternal forces of labor (ie, uterine contractions, maternal pushing) far exceed physician traction forces and are the putative cause of permanent brachial plexus injury in the majority of cases. Several lines of evidence confirm this assessment.

Gonik et al, using sophisticated mathematical modeling, demonstrated that maternal forces of labor are twofold to fourfold that of physician traction.^39–41^ Complementing the modeling studies, an array of clinical and experimental studies have confirmed these relative values. Briefly, when intrauterine pressure is converted to a measure of force (on the presenting part), uterine contractions in the first stage of labor result in approximately 25 foot-pounds. During the second stage of labor, maternal pushing during contractions results in a force of approximately 50 foot-pounds. With the positioning of McRoberts maneuver, there is a nearly twofold increase in intrauterine pressure and, thus, a resulting force of approximately 100 foot-pounds.^42^ Although McRoberts efficacy has been attributed to the opening of the pelvic angle, the significant increase in maternal forces also likely contributes to shoulder delivery. In contrast to maternal forces, physician traction forces have been quantified as 25–30 foot-pounds, confirming the modeling assessments of Gonik et al. Because force equals pressure×surface area (ie, fetal presenting part), maternal force values are somewhat greater in larger fetuses, perhaps contributing to the increased rate of brachial plexus injury in macrosomic neonates. Though clinician traction forces can be assessed only by the clinician as opposed to observers, it is notable that both obstetric residents and faculty are excellent judges of applied traction force.^43^

How then does one explain the finding that only 4–23% of deliveries involving shoulder dystocia result in an initial brachial plexus injury and that fewer than 35% of brachial plexus injuries are permanent?^44^ Rather than attributing these cases to excessive clinician traction, individual maternal or neonatal variability likely contributes. Firstly, brachial plexus anatomy varies significantly among individuals,^45^ potentially altering the focal points of stretch. Secondly, consistent with the evidence of developmental origins of health and disease, the development of supporting bone,^46^ muscle,^47^ and nerve fiber^48^ varies depending on both fetal genetics, epigenetics and the maternal–placental environment.

There have been extensive efforts by ACOG and others to improve the identification of shoulder dystocia, the steps of management (eg, elimination of fundal pressure), and the institution of simulation training. Yet, the overall rate of brachial plexus injury has not significantly decreased. Together with the evidence noted above, these findings indicate that brachial plexus injury is primarily an intrinsic risk of labor, both with and without shoulder dystocia.^49^ Although it is clear that excessive traction can cause brachial plexus injury, to claim de facto that permanent brachial plexus injury resulting from a shoulder dystocia delivery must be a consequence of excessive traction is simply junk science.

## Tachysystole and Elevated Uterine Resting Tone Result in Fetal Compromise

Among the uterine contraction descriptors (basal tone, Montevideo units, frequency, duration, resting period), only the frequency (ie, tachysystole) has been identified and quantified by ACOG as potentially concerning. Whether assessed with intrauterine pressure catheters or external tocodynamometers, ACOG defines *tachysystole* as more than five contractions in 10 minutes, averaged over 30 minutes, thus equating to 16 (or perhaps 15.5) contractions in 30 minutes.^50^ Smith et al^51^ reported periods of tachysystole occur in approximately18% of term labor cases, and fetuses with neonatal depression or metabolic acidemia did not have more tachysystole than those without. Among patients induced with oxytocin, Kunz et al^52^ reported that 98% demonstrated one or more occurrences of tachysystole; patients who received misoprostol cervical ripening or induction often exhibit tachysystole.^53^ Although increased uterine contraction frequency may be associated with an increased risk of fetal hypoxia^54^ and acidosis at birth,^55^ the fetal effect is largely dependent on fetal heart rate responses evidenced on the tracing.^56^ A recent systematic review concluded that the evidence that increased uterine activity may predict depressed newborn neurologic function is inconsistent and insufficient.^57^

Uterine resting tone has been alleged to be associated with clinician negligence and the cause of hypoxic brain injury. *Resting tone*, which can be quantified only with an intrauterine pressure catheter, is defined as the basal tone between contractions. The concern with elevated basal tone arose from experience with placental abruptions in which high frequency uterine contractions (8/10 minutes) prevented uterine relaxation.^58^ Based on results from fluid-filled intrauterine pressure catheters, a basal value of 25 mm Hg has been commonly accepted as a normal upper limit. Fluid-filled intrauterine pressure catheters use a pressure sensor that is leveled to the top of the maternal fundus. Intrauterine pressure measurements are a composite of the pressure generated by uterine tone, intra-abdominal pressure, and the effect of amniotic fluid, placenta and fetal mass, and maternal tissue. Both tissue and fluid result in a pressure of approximately 0.74 mm Hg per cm of vertical height. Thus, based on fluid physics, the placement of the catheter tip below the level of the transducer results in a reduction in the net uterine tone and intra-abdominal pressure by an amount to cm (distance)×0.74.

The concept of a uterine resting tone threshold of 25 mm Hg is an example of misinformation. With the introduction of transducer tipped intrauterine pressure catheters, pressure measurement values have changed. The transducer tipped intrauterine pressure catheter has a hollow catheter that zeros the internally placed transducer to room air. Thus, the transducer tipped intrauterine pressure catheter measures both the uterine tone and intra-abdominal pressure as well as the vertical height of amniotic fluid, and fetal, placental, and maternal mass above the catheter. When inserted, intrauterine pressure catheters are typically directed anteriorly. With progress in labor and gravitational forces, the intrauterine pressure catheter commonly shifts to the lower area of the uterus, resulting in a falsely elevated basal tone, due to the greater height of the amniotic fluid or fetal column.^59^ With a uterine height of 20 cm, a shift from anterior to posterior within the uterus may increase basal resting pressure by 15 mm Hg. If one assumes that both the fluid-filled intrauterine pressure catheter and the transducer tipped intrauterine pressure catheter are similarly situated at the inferior portion of the uterus, the basal uterine tone values may differ by 30 mm Hg, because the fluid-filled intrauterine pressure catheter will measure 15 mm Hg below the actual pressure (assuming the pressure transducer is appropriately positioned at the top of the fundus) and the transducer tipped intrauterine pressure catheter measure 15 mm Hg above the actual pressure. Because both resting pressure and peak uterine pressures are affected equally, Montevideo units are reliable as a measure of contractile strength. Thus, elevated basal uterine tone in the absence of a placental abruption is typically an artifact resulting from use of transducer tipped intrauterine pressure catheters and generally is not associated with fetal compromise in the absence of persistent tachysystole.

## DISCUSSION

The scope and effects of obstetric malpractice claims are notable. A nationwide survey using the Jury Verdict and Settlement Analyzer through Lexis Nexis under the terms “fetal” and “hypoxia” returned 2,151 cases, of which 735 are reported as settlement, 368 plaintiff verdicts, and 274 defendant verdicts with average recoveries of $3.38 million, $19.0 million, and $0, respectively. When searched for “fetal” and “brachial plexus,” Lexis Nexis reported 1,110 cases, of which 271 were reported as settlement, 162 as plaintiff verdicts, and 214 as defendant verdicts with average recoveries of $1.2 million, $4.1 million, and $0, respectively. Notably, there are occasional settlements that require the settlement details to be confidential and various laws that can affect verdict values, which, in turn, may affect recovery. These values underscore the level of settlements and judgments intrinsic to obstetric malpractice litigation. The approximate fourfold dollar value for plaintiff verdicts as compared with settlements potentially may incentivize the plaintiff to pursue litigation. With anticipation of these recovery rates, arguments of junk science, unfortunately, may be used.

Proponents of junk science have adversely affected obstetric and gynecologic clinical practice with allegations of fault when none may exist.^60^ Specifically, malpractice allegations have resulted in the withdrawal of drugs from the market (eg, Bendectin), lower rates of instrument deliveries, and a markedly increased rate of cesarean delivery.^1,2^ The scientific principles of medical causation should be the basis to establish a causal relation between a specific factor and an adverse outcome. Among these principles include biological plausibility, in which an understanding of physiology, biochemistry, and pathology establishes a credible causative pathway.

Herein, among the clinical scenarios of uterine contraction frequency and resting tone, physician traction forces, and fetal compromise, credible scientific data, and physiologic principles are presented. In contrast, junk science may be based on poor-quality studies (eg, not controlled, post hoc analysis, biased data, no proof of causality).^6^ Although a strong association between a risk factor and an outcome is more likely to suggest a relationship within a causal pathway, it does not prove causation. One may consider the use of relative risks or odds ratios to establish whether, based on the risk factor alone, the outcome is “more likely than not” to be associated with the risk factor. As discussed above, a temporal relationship must demonstrate that a cause precedes the outcome, though this alone is insufficient to prove causality. As a biological mechanism, scientific principles generally demand establishment of dose, intensity, and duration versus response relationship, which, again, supports a causality mechanism but does not prove one. The use of experimental studies with control cases, adjusting for confounding variables, determining sensitivity and specificities of association, and dose response relationships are ideal for scientific acceptance. Animal studies may provide additional mechanistic insights. However, for many issues in obstetrics, animal models may not be optimal, and controlled human experimental studies cannot be performed. Accordingly, this has led, in part, to the advent of junk science proposals filling a gap.

The courts have partially addressed issues of junk science with the use of Daubert and Frye hearings,^61,62^ though it is a complex challenge for a judge, typically not trained in science or clinical medicine, to differentiate misinformation or junk science from established scientific principles.^63^ Similarly, it is all the more challenging for a jury of laypersons to differentiate. There are numerous other examples of junk science in obstetric litigation, including allegations that contraction-induced fetal head compression causes brain injury in the absence of fetal systemic acidosis, newborn “acid washout” after delivery, adverse effects of shortened uterine resting time, and a plethora of alleged pharmaceutical-induced teratogenesis (eg, Bendectin). It is important to emphasize that scientifically credible and valid evidence be presented by both plaintiff and defense positions.

It is commendable that societies such as the Society for Maternal-Fetal Medicine and ACOG publish guidelines that outline algorithms of care. With these examples of junk science detailed above, it would be of value for medical societies to expand the content of opinion and position statements to include assessments of science, such that it can aid in adjudicating malpractice allegations and preventing unsubstantiated alterations in clinical management.
